# Mothers’ Resilience: Experiences of Intimate Partner Violence Survivors at Work

**DOI:** 10.3390/children9030373

**Published:** 2022-03-08

**Authors:** Kathryn Showalter, Kathryn Maguire-Jack, Rebecca McCloskey

**Affiliations:** 1 College of Social Work, University of Kentucky, Lexington, KY 40508, USA; 2 School of Social Work, University of Michigan, Ann Arbor, MI 48109, USA; kmjack@umich.edu; 3 Mighty Crow Media, LLC, Columbus, OH 43214, USA; rebecca@mightycrowmedia.com

**Keywords:** intimate partner violence, qualitative research, mothers, employment

## Abstract

Mothers who experience intimate partner violence can be resilient in maintaining employment during periods of abuse. The current qualitative study examines mothers’ experiences of abusive workplace disruptions as well as helpful responses from workplaces. Two main research questions are addressed: 1. What ways do abusive partners use issues related to children to disrupt mothers’ employment? 2. How do workplaces respond to mothers experiencing IPV? How do mothers show resilience? Mothers (*n* = 18) receiving services for abuse explained that abusive partners disrupted their work through compromising or withholding childcare, manipulating them through children, and jeopardizing child safety during work hours. However, mothers showed resilience when coworkers extend housing, childcare, and genuine concern for their situations. Implications for researchers, practitioners, and employers of survivors are discussed.

## 1. Introduction

Employment instability or loss of paid work time and unemployment persists for weeks, months, and years in the lives of intimate partner violence (IPV) survivors in the United States [1,2,3]. In a longitudinal study of the effect of IPV on employment trajectories, researchers found that experiencing IPV relates to unemployment six years after abuse occurred among mothers [4]. IPV survivors who are mothers may be more prone to experiencing unemployment. The added responsibility of parenthood and manipulation from abusers to prioritize children over work can lead mothers to leave their jobs [5,6,7]. However, previous research indicates that mothers who feel supported in the workplace may show resiliency and continue to stay employed despite abuser efforts to sabotage them [2,6]. The current study expands understanding of mothers’ employment instability by using a qualitative approach to clarify the effect of parenting-specific abusive workplace disruptions on employment status and the potential protective effect of workplace support from coworkers.

### Literature Review

There are various typologies of IPV including forms of abuse, types of violence, and types of perpetrators [8]. The forms of abuse include physical violence, sexual violence, and psychological violence. The types of violence examine the patterns within which the violence occurs, while the types of perpetrators typology focus on factors about the perpetrators themselves [8]. In the current study, we examine a slightly different typology of IPV, in which we are examining the specific situations (work) in which abuse occurs, and specific tools (childcare and children) that abusers use to commit violence.

Abusive workplace disruptions are tactics used by abusive partners to prevent survivors from attending work and performing to their full potential. In the context of parenthood, abusive workplace disruptions include excessive contacting about children (40.6%), sabotaging childcare arrangements (38.0%), and having to take time off work due to child-custody disputes (22.5%) [9,10]. Abuser-initiated workplace disruptions are closely related to survivors’ employment instability [3,11,12]. About half of survivors (*n* = 133) in a study of women experiencing abuse in the last 12 months reported that they were reprimanded or lost their job during abuse periods [13]. Workplace disruptions also lead to employment instability through indirect pathways. In a Banyard and colleagues [14] study of women living in New Hampshire (*n* = 1079), survivors reported problems concentrating at work, working at a slower pace, and feeling exhausted at work significantly more often than women who did not experience IPV.

Motherhood poses an opportunity for abusive partners to confound victims’ employment and financial stability. Several previous studies with samples of mothers find that IPV is significantly related and negatively impacts employment outcomes [3,11,15,16,17,18,19]. In a study comparing abusive workplace disruption tactics across multiple samples, 38% of survivors reported experiencing childcare threats and 11% reported that a partner told them “women shouldn’t work outside the home” or “women who work outside the home are bad mothers” [9] (p. 749). Researchers analyzing abusive workplace disruptions with item response theory found that having childcare arrangements purposely sabotaged by abusers was a common experience among survivors [19]. Thus, employment instability is likely especially common among mothers.

Working parents face significant challenges in the United States because of the availability, hours, quality, and cost of childcare [20]. Working mothers who are survivors of IPV face additional challenges due to their experience of IPV. The high cost of childcare may be factored into a mother’s decision to leave an abusive partner; that is, to the extent that the abusive partner contributes to the overall finances of the home, the survivor may be unable to separate themselves from the partner because of the inability to afford childcare on their own. Across multiple studies, survivors report that childcare assistance is a significant need [21,22].

Mothers who are IPV survivors may be reluctant to leave their children home with their abuser, for fear that the abusive partner will harm the children when the mother is not home to protect them [7]. Children of parents experiencing IPV are at significantly greater risk for child maltreatment [23]. In homes where IPV is present, children are 2.5 times more likely to experience physical abuse and 9.5 times more likely to experience psychological abuse [24].

Several researchers have explored employment experiences of IPV survivors [25,26] and about 35 known published papers have investigated survivors’ employment using qualitative approaches previously (see [27]). Of these, eight focus on aspects of motherhood, intimate partner violence, and employment but none of them focus on mothers’ resilience at work. In a related qualitative study utilizing the same data as the current study, but that did not focus on motherhood or resilience, subjects reported leaving work when abusive partners left young children home alone, missing work to help children emotionally recover after IPV incidents, and being unable to provide for children without employment [28]. The current study addresses the following research questions:

1. What ways do abusive partners use issues related to children to disrupt mothers’ employment?
2. How do workplaces respond to mothers experiencing IPV? How do mothers show resilience?

## 2. Materials and Methods

This study was approved by the Institutional Review Board (IRB) of a large midwestern university and was given full consideration. The research utilized a sample of 19 clients receiving counseling services for IPV at a Midwest social services agency from 2017 to 2018. A convenience sample of participants were recruited by agency counselors by phone and in person if they met the following eligibility criteria: 18 years of age or older, English-speaking, identify as female, and currently or previously employed while experiencing IPV. Sampling was determined to be complete when saturation was reached and additional interviews did not provide further insight into survivors’ employment instability [29]. The current sample excludes one participant who did not have children.

If agency clients agreed to participate, an interview time was made by the counselor after their regularly scheduled counseling session in a private room at the agency or in a public library. The first author, a White female, who had no personal experience with IPV, conducted all semi-structured interviews. In an exercise of researcher reflexibility, the author recognized her position as both an outsider and an educated expert. She utilized engagement and interview skills obtained through her social work education to establish rapport and positively influence the relationship between interviewer and participant. All conversations started by obtaining verbal consent, agreement to record the interview, and answering any participant questions. During the subsequent 45–65 min conversations, participants responded to demographic questions and approximately 15 open-ended questions related to their experiences of employment instability. Participants received a USD 20 incentive for their time and travel.

### Analysis

Audio data were transcribed by professionals approved by the IRB and uploaded to NVivo Pro 12 for Windows for analysis [30]. Two coders were used to analyze the data to enrich the quality of analysis and to avoid individual researcher bias. A constructivist paradigm framed the analysis whereby the researchers served to interpret participants’ unique realities which emerge from their individual experiences and life contexts [31]. As guided by grounded theory experts [32], the following coding steps included (1) reading transcripts carefully, (2) open coding, (3) axial coding (grouping codes into categories), and, last, (4) selective coding and comparison of categories. Throughout completion of the independent coding of the data, the first and third/fourth authors met weekly to discuss codes, potential themes, and their interpretations, and questions that arose during analysis. Additionally, coding memos and an audit trail were maintained to ensure further trustworthiness of the study [33]. The final selection of themes was made in consultation after resolving the few areas where suggested themes differed.

## 3. Results

Descriptive statistics illustrate a homogenous sample. Interview participants had an average age of 38 (SD = 10.76) and a majority had more than a high school education (66%). Approximately two thirds of interview participants identified as White and about 11% identified as Hispanic. Participants on average had two children.

Using research questions as guidance, four major thematic categories were identified: childcare challenges; manipulation through children and parenting; child safety and wellbeing; workplace supports. Within themes, quotes from participants offer insight into mothers’ experiences. See Table 1 for complete descriptions of themes.

### 3.1. Childcare Challenges

Approximately one-third of the sample reported that abusive partners used childcare as a means to make participants late to work and/or leave work early. One participant, employed as a nursing professional, explained “I had to call off for the kids when the kids were under stress… It was just a lot of call offs and a lot of tardiness”.

Survivors expressed that abusers did not see transporting children or taking care of them during work hours as their responsibility, not even on rare occasions. One survivor explained

“I would be all ready to go, and he [abusive partner] would be lying in bed. He would say, ‘Where the hell are you going?’ I’m like, ‘Okay. Well, this is the day that I have to be at work at 6:00…’. He goes, ‘I’m not taking those effing kids to daycare”.

Further, another mother who had a long commute stated she would have to pay late fees to childcare providers for not picking up the children on time:

“It was a constant stress thinking about the traffic home, and driving on the highway, and not knowing whether it was going to go or stop. Just knowing that I would not be able to call him and say, ‘Hey, I’m stuck in traffic, will you please pick up the kids?*’.* I ended up paying extra sometimes for them at daycare’”.

### 3.2. Manipulation through Children and Parenting

Still, about one in four participants recalled that they were not able to work at all because of childcare responsibilities. Abusive partners prevented mothers from working by stating that childcare was too expensive or that the children would suffer if mothers/both parents went to work. One survivor recalled the excuses she received from her partner:

“Well, you can’t work, we can’t afford daycare, the kids will miss you, and what are you gonna do with the kids?”.

Additionally, one participant experienced prolonged isolation and financial abuse that resulted from not working or utilizing childcare:

“For years I was a stay-at-home mom because he [partner] didn’t want me to work. He wanted to take care of me and wanted me to do the motherly duties, but then I had no access to funds. I had no right to know how much money he was making. It was none of my business”.

Abusive partners even manipulated children to call their mothers and beg them to leave work. One survivor explained her suspicion when her young child left her a voicemail: “Mommy, I really miss you. Can you please come home? I’m going, ‘You’re three years old. You do not do this on your own’”.

To exacerbate situations, abuse partners continued to use children to manipulate and scare mothers even after they separated. One survivor recalled her abusive partner ruined her confidence to provide for her children by saying “You’ll never get custody. You’re a bad mom, you don’t have a job, you don’t have this and that”.

### 3.3. Child Safety and Well-Being

As primary caregivers, participants who experience violence in their homes struggle to keep their children safe. Concerns of children’s safety and well-being was mentioned by about 20% of participants. One survivor explained the difficulty of lying to coworkers to keep her daughter safe. She told her coworkers:

“‘Well, I’ve gotta pick my daughter up. I don’t have nobody to watch her’, and it was just like that wasn’t the truth. I had somebody to watch her, but I didn’t trust where he [abuser] was gonna take her cuz he has something’ to do, but it wasn’t work. It was whatever he wanted to do”.

Other survivors had abusive partners that threatened harm or neglect to children if mothers did not leave work. One survivor with two young children remembered her partner’s frightening words and actions:

“‘You leave, I’m gonna leave the kid.’ I’m like, ‘You’re not gonna leave my kid. I have to go back to work.’ No, he left my kids home alone so I had to go back”.

Similarly, survivors were afraid that if they did not leave work that abusers might kidnap or take their children. This fear was present for one survivor:

“I just was to the point where I was just so scared like, where is he gonna take my baby? Then I get off work and not know where she’s at. I would leave early cuz I’m like, ‘I gotta get my baby’”.

### 3.4. Workplace Supports

Coworkers, managers, and employers did offer support to mothers who were experiencing IPV. Specifically, supports included watching children, talking through safety plans, offering a safe place to stay, and regularly checking in. These supports were reported by about 30% of the sample and contributed to the resilience of mothers in terms of continuing to work through periods of abuse.

Some coworkers took a very active role in protecting mothers and their children. One participant recalled help from her supervisor: “She offered actually for a place for me to stay. Like for me and my kids to move in with her until I figure things out”. Additionally, a participant mentioned that her coworker would watch her children on days off, not charging the survivor for babysitting.

More commonly however, survivors shared that coworkers would help de-escalate stressful situations involving abusive partners and check in with survivors. One mother stated

“Honestly, she [coworker] just listened and she wanted to make sure that I was safe and that my daughter was safe. We only had one kid the first time around. She really just did a lot of regular check ins”.

## 4. Discussion

Previous research has found irregularity in the effect of IPV on employment outcomes as well as the longevity of employment instability for survivors [1,17]. The current study sought to explore the ways in which abusers use children to sabotage employment among mothers as well as to understand how mothers show resilience with workplace support. We found that IPV among mothers causes unique challenges that impacts their ability to work.

While childcare is a concern for all working parents, for survivors this is even more challenging [19]. Mothers in the current study reported that their abusive partner was unwilling to assist with logistics of childcare in any way. Childcare centers typically follow standard work schedules and charge exorbitant fees to parents who are late to pick up their children. Participants in this study emphasized how difficult it was to meet their required work schedules while also meeting the requirements of childcare drop-off and pick-up. Mothers in the study sometimes had to weigh the demands of these two systems, having to decide whether they could afford to leave work early and face the consequences of the employer, or afford the fees imposed by the childcare center.

Within the typologies of IPV examined for the current study, we found that abusers use childcare as a tool to commit violence against their partners. Specifically, the study found that abusers use childcare as a justification to prevent mothers from working at all, pointing to the high cost of childcare as referenced in previous literature [20]. In doing so, they limit the survivor’s contact with others who may be supportive to them and make them financially dependent on the abuser. Financial abuse is a significant problem within the context of IPV. In a review of quantitative studies (*n* = 46), researchers found that most studies only ask one or two questions to survivors about financial abuse and so it is difficult to determine prevalence, but financial abuse is often correlated with physical and psychological abuse [32]. Additionally, survivors who are not working are less able to leave the violent relationship.

We found that abusers also use threatened or implied violence or neglect of children in order to wield power and control over their partners. Mothers reported fearing leaving their children home with the abusive partner, which supports previous research suggesting that this is a problem for working mothers who are survivors [7]. Because child maltreatment and IPV commonly co-occur [23,24], there is a significant risk to children. In addition to concern about violence against the children, mothers also reported abusers threatening to kidnap children or refusing to supervise children. In addition to the harm caused to mothers, the violence and threat of violence to children is also harmful to child development. This finding builds on related literature [12,15,16,17,28] by illustrating that survivors are solely responsible for the safety of their children.

Abusers used the children themselves as a tool against the mother while she was working. Mothers reported the child calling them while they were at work to tell them that they missed them and wanted them to come home. This manipulation may contribute to mothers leaving work early, being distracted at work, or even experiencing disciplinary action at work for receiving too many personal phone calls. Interestingly, in contrast to a previous qualitative study of mothers, there was no mention of substance abuse from abusive partners around children being a concern [25].

Although the IPV situation contributed to stress and disruptions to employment, mothers in the sample displayed resilience. They were committed to keeping their children safe and worked hard to maintain their employment, despite challenging circumstances. Some mothers reported that their workplaces provided supports to them that were helpful. Coworkers and supervisors talked through safety plans with survivors, offered them a place to stay, and provided a listening ear to them. There was not mention of other survivors in the workplace recognizing signs of IPV and stepping in, as has been reported in related literature [25,28]. Workplaces can support survivors further by providing education and training to employees about IPV and how to support each other. Informally, coworkers have been found to counter abusive behavior by showing concern, asking if survivors are okay/safe, or offering to make phone calls to access resources [28].

### 4.1. Limitations

The current study expands upon employment instability concepts and addresses questions of maternal caregiving and financial stability during intimate partner violence experiences. However, limitations of the current study exist within the data source and analysis. Interview data is limited because it is not representative of all survivors’ employment experiences. Specifically, the sample reflects a group of women who volunteered to participate, had some type of education after high school, largely identified as White and non-Hispanic, experienced IPV requiring professional treatment, and volunteered to participate in the study. Further, both researchers of the data may have exerted bias during the coding process based on feminist ideals and individual privileges.

### 4.2. Implications

Employers have a responsibility to protect employees from harm during work hours and that includes IPV. Workplaces that help survivors work consistently over time not only financially protect survivors but also avoid costs of employee turnover and absence. Given qualitative findings, survivors likely need time off from work to physically and emotionally recover from abuse as well as to provide for their children. If policies such as the Family Medical Leave Act (ACT) could be adapted to cover incidents of IPV, survivors could take needed time off from work without risk of being fired and losing their families’ financial livelihood.

Research on the interventions for employment instability among IPV survivors is needed. To start, the employment services offered to IPV survivors are largely focused on financial well-being, such as Moving Ahead through Financial Management or asset building with Individual Development Accounts [34]. However, these programs are not focused on employment. While one known evidence-based practice exists (i.e., ACCESS; Advancing Career Counseling and Employment Support for Survivors of Domestic Violence) [35], it is limited in generalizability and accessibility. Further, none of these interventions focus on mothers who are struggling to care for their children. As researchers continue to develop interventions and sector-specific support, we recommend that service providers support mothers at work by utilizing safety planning or helping them to enforce state-level protections [36].

## 5. Conclusions

Clarifying mothers’ employment experiences and resiliency while enduring partner abuse is a significant contribution of the study. Childcare, manipulation through children, and child safety emerged as significant concerns of women who experienced IPV. Thus, at the practitioner level, advocates need to implement workplace safety planning and connect survivors with human resource services. Further, policymakers need to extend housing and childcare funding to mothers experiencing intimate partner violence as they are key in keeping survivors working. Last, instead of treating IPV as a “private matter”, employers need to take responsibility for employees’ safety and wellbeing so that women may gain financial independence from abusive partners.

## Figures and Tables

**Table 1:** Themes, theme definitions, and codes.

Themes	Theme Definitions	Codes
Childcare Challenges	Abusive partners use childcare as a way to force survivors to leave work to take care of their children.	Transportation, leave work, parenting disruption.
Manipulation through Children and Parenting	Maternal responsibilities were used to make mothers feel guilty about going to work.	Guilt, maternal duty, parenting ability.
Child Safety and Wellbeing	Children were put at risk by abusive partners through threats of kidnapping or abandonment.	Danger, maternal fear, kidnapping.
Workplace Supports	Coworkers offered physical and emotional supports to mothers.	Childcare assistance, housing, listening, checking in.

## Data Availability

The data presented in this study are available on request from the corresponding author. The data are not publicly available due to the risk of identification.
